# Necrology

**Published:** 1897-02

**Authors:** 


NECROLOGY.

William Tag, Ph.G., V.M.D., graduate of the Veterinary Department of the University of Pennsylvania, class of '91, died at Philadelphia on January 12, 1897. Dr. Tag had secured in the northern district of Philadelphia a growing clientage which promised much for him in his professional career.

